# Factors associated with COVID-19 fatality among patients admitted in Mashonaland West Province, Zimbabwe 2020-2022: a secondary data analysis

**DOI:** 10.11604/pamj.2023.44.142.37858

**Published:** 2023-03-21

**Authors:** Kudzai Madamombe, Gerald Shambira, Gift Masoja, Tapiwa Dhliwayo, Tsitsi Patience Juru, Notion Tafara Gombe, Addmore Chadambuka, Mujinga Karakadzai, Mufuta Tshimanga

**Affiliations:** 1 Department of Primary Health Care Sciences, Family Medicine, Global and Public Health Unit, University of Zimbabwe, Harare, Zimbabwe,; 2 Zimbabwe Ministry of Health and Child Care, Mashonaland West, Zimbabwe,; 3 Africa Field Epidemiology Network, Harare, Zimbabwe,; 4 Zimbabwe Community Health Intervention Research, Harare, Zimbabwe

**Keywords:** COVID-19, mortality, isolation center

## Abstract

**Introduction:**

approximately 15% of COVID-19 patients develop symptoms necessitating admission. From 2020 to 2022, Mashonaland West Province had an institutional case fatality rate of 23% against a national rate of 7%. Therefore, we evaluated the COVID-19 admissions in the province to determine the factors associated with COVID-19 mortality.

**Methods:**

we conducted an analytical cross-sectional study based on secondary data from isolation centers across the province using all 672 death audit forms and patient records. We obtained data on patient demographics, signs and symptoms, clinical management and oxygen therapy administered, among other things. Data were entered into an electronic form and imported into Epi-info 7 for analysis bivariate and multivariate conducted. **Results:** we found that being an older man, aOR 1.04 (1.03-1.05), who had diabetes aOR 6.0 (95% CI: 3.8-9.2) and hypertension aOR 4.5 (95% CI: 2.8-6.5) were independent risk factors. Patients put on dexamethasone aOR 2.4 (95% CI: 1.6-3.4) and heparin/clexane aOR 1.6 (95% CI: 1.1-2.2) had a higher mortality risk. However, vitamin C aOR 0.48 (95% CI: 0.31-0.71) and oxygen therapy aOR 0.14 (95% CI: 0.10-0.19) and being pregnant aOR 0.06 (95% CI: 0.02-0.14) were protective. **Conclusion:** mortality risk increased in older male patients with comorbidities and with those on dexamethasone and heparin therapy. Oxygen therapy and vitamin C were protective. There is a need to conduct further study of the source of these variations in risk across patients to establish the true impact of differences in individuals' mortality.

## Introduction

The Coronavirus Disease of 2019 (COVID-19) [1] is a contagious disease caused by a unique coronavirus [2] that has spread around the world, including Zimbabwe. According to the World Health Organization (WHO), the virus has infected over 380 million individuals and killed more than 5 million people [3]. The virus spreads quickly from person to person, with one individual being able to infect up to three people [4] and approximately 85 percent of COVID-19 cases are thought to be asymptomatic [5], whereas 15 percent develop symptoms that may necessitate admission to an isolation unit [6]. Asymptomatic, mild, moderate, and severe diseases are the clinical stages of COVID-19 infection [7]. Patients who test positive for COVID-19 but do not have any symptoms suggestive of the condition are asymptomatic. Patients with moderate disease have any COVID-19 signs and symptoms (fever, cough, sore throat, malaise, headache, muscle pain, nausea, vomiting, diarrhea, loss of taste and smell), but no shortness of breath, dyspnea, or abnormal chest imaging. Patients with moderate disease have an oxygen saturation (SpO_2_) of less than 94 percent on room air and have signs and symptoms of lower respiratory disease. Oxygen saturation (SpO_2_) 94 percent on room air, a respiratory rate greater than 30 or respiratory failure, septic shock, or multiple organ dysfunction are all considered severe illnesses [8]. The virus's fast spread significantly strains many countries' public health systems [5], sparking a massive demand for hospital services that were not always provided [9]. According to estimates, 3% [10] and 32% [6] of patients with COVID-19 symptoms were admitted to the hospital globally. Because of the tremendous demand, some European and American hospitals have begun to report an in-hospital mortality rate as high as 86 percent [10]. The elderly and persons with comorbidities, who are the most vulnerable elements of the population, have been disproportionately targeted [2].

In Africa, things were vastly different. As of January 2022, the WHO reported that 8.1 million people in Africa have contracted the virus, with over 40 thousand deaths [3]. Although the continent was home to approximately 14% of the world's population, only 4% of the overall number of COVID-19 cases and about 3% of the deaths were reported [11]. However, it's worth noting that Africa is the world's youngest continent, which may help explain the gap [12]. Though, obtaining reliable statistics on African hospital admissions and, as a result, data on inpatient mortality might be complex [13]. In Africa, Zimbabwe has the eighth-highest number of COVID-19 cases [3]. Over 220 thousand COVID-19 infections have been reported in the country, with 5350 people dying as a result of the epidemic [14]. The Ministry of Health and Child Care (MOHCC) set up isolation centers in each province to accommodate all COVID-19 patients who require admission. Three isolation centers were established in Mashonaland West Province: Chinhoyi Provincial, Karoi General, and Queen Mary. In addition, due to the high demand for hospital beds the districts identified 11 other sites and established isolation centers bringing the total sites to 14. Mashonaland West Province had a very high COVID-19 institutional case fatality rate of 26%. This is against the national institutional fatality rate of 7%. The admitted cases in the province increased from 32 to 605 patients between May 31^st^, 2021 and February 21^st^, 2022. The case fatality rate was 19% (141) out of the 605 patients. The province took several steps to address this, including providing comprehensive COVID-19 case management training to all healthcare professionals working in isolation centers, outfitting all isolation facilities with ventilators, and creating a COVID-19 audit form. However, the audit forms and patient records data were yet to be analyzed. Analysis of this data will help highlight the areas in the province that performed well whilst identifying the less-than-optimal areas for future improvement in potential upcoming waves. Therefore, we collected all the audit forms and patient records and analyzed the COVID-19 data to describe admitted patients' deaths, determine the specific characteristics associated with death or survival, and estimate the survival time.

**Operation of the audit:** to track every admitted COVID-19 patient, the province created a paper-based audit form. After the patient was discharged, nurses at isolation centers entered data into the audit form. Following that, copies are sent to the District Health Information Officers. The audit form collected the following variables: age, sex, isolation center, disease severity, referral status, pre-existing conditions and pregnancy-related conditions, type of management administered, cause of death, and place of death among others. The audit forms aim to collect COVID-19 patient data to improve inpatient care quality and prevent future deaths.

## Methods

**Study design:** an analytic cross-sectional study based on secondary data was conducted to determine the factors associated with institutional fatality from April 2020 to April 2022 in Mashonaland West Province.

**Study setting:** our research setting was the 14 isolation centres in Mashonaland West. The population of Mashonaland West Province is 1,989,399 people (DHIS estimates 2021). Chegutu, Hurungwe, Kariba, Makonde, Mhondoro-Ngezi, Sanyati, and Zvimba are the province's seven districts. Emergency preparedness and response (EPR) teams and rapid response teams in the districts coordinate COVID-19 operations (RRTs). In the seven districts, there are a total of 365 beds accessible for COVID-19 patients. However, only 51 of the 365 (14%) beds have access to oxygen, and only six operational ventilators exist. In the province, there are 14 isolation centers. In its isolation centers, the province has admitted 672 cases.

**Study participants and sampling:** we conducted a census of all records available from April 2020-April 2022. Our study population included all available COVID-19 death audit forms and all available patient records.

**Study variables:** we measured the variables such as the patient's age, time of admission, duration of symptoms at home, comorbid conditions present, the length of therapy until the patient was discharged or died, and reporting district were all gathered using COVID-19 death audit forms. The time between ordering a procedure or treatment and its actual administration was measured using patient notes and ward registers to determine institutional delays. The reasons for the high fatality rate were investigated by interviewing key sources such as medical staff and hospital executives. The dependent variable for our study was the patient outcome on the audit form. The two outcomes were whether the patient survived or whether the patient died. We assessed the sociodemographic data by extracting data on age, sex, vaccination status, vaccine type, and district of origin. We evaluated COVID-19 vaccination by extracting data on vaccination status, type of vaccine received, and the number of doses received. We evaluated the signs and symptoms by extracting data on the patient history of presenting complaints, social history, including drinking habits, smoking habits, water and sanitation history, and past medical and surgical history. Finally, we assessed the examination findings at admission. Comorbidities were assessed by extracting data on the type of comorbidity the patient had at the time of admission. The management given was evaluated by extracting data on investigations done, daily vitals monitoring, daily review of management given, differential diagnosis, discharge notes, oxygen therapy, and intensive care unit. We assessed the duration of admission by extracting the date of admission and date of discharge and calculating the difference between the two.

**Data capturing and analysis:** we extracted data from patient audit forms and patients' records and entered it into an electronic google sheet form adopted and adapted from WHO COVID-19 case audit forms [15-17]. The data was collected in comma-separated values (CSV) format and then imported into R statistical software before being analyzed with Epi-Info 7. We checked for missing values, and variables with greater than 5% missing data were handled using a multiple imputation method. Rubin's guidelines [18] were used to aggregate the results of five imputations. The age, duration of admission and the delay in seeking care continuous numerical variables were tested for normalcy using the histogram method and the Student t-test and were found to be not normally distributed. The categorical variables were provided as frequencies and percentages, whereas the findings of age were presented as median (interquartile range, IQR). The distinct patterns of COVID-19 deaths in Mashonaland West Province and their statistical significance were visualized using trends, confidence intervals, and p-values. The variables examined were the number of COVID-19 deaths by district, prevalent comorbid diseases, signs and symptoms upon presentation, patient management, and length of stay at the time of death. We used univariate and bivariate analysis on each statistically significant variable. Then, we used multivariate analysis on each variable one at a time to find all independent determinants for COVID-19 survival and mortality among hospitalized patients in Mashonaland West.

**Ethical considerations and permission to proceed:** the Mashonaland West Provincial Ethics Committee reviewed and approved the study. We did not record the names of medical personnel and patients admitted to isolation centers. Instead, an anonymous identification number was assigned to each audit form and patient record during data collection and processing. Confidentiality was maintained in interviews with key informants, no names were recorded on the questionnaire, and the physical questionnaires were kept under lock and key. The Provincial Medical Director Mashonaland West Province, District Medical Officers, and the Health Studies Office, all permitted us to proceed. The study's findings were communicated with Mashonaland West's isolation centers, medical staff, and the Provincial Health Executive (PHE) of Mashonaland West.

## Results

**Data quality:** the data quality was summarized as follows. Out of the 672 records, 96% had all the demographic details of name, age, sex and address and the duration of stay was recorded in all the records. Ninety-nine percent of the records had the presenting complaint(s) documented. Only 65% of the records had examination details and laboratory tests documented. The medical history was recorded in 75% of the records, while 80% had a social history. The patient outcomes (whether the patient was discharged or deceased) was recorded in all the records.

**Patient demographics:** the province admitted 673 patients into isolation centers. We found that most patients were female 446 (66.4%) and the median age was 40 years (Q1=25, Q3=65). Out of the deceased (n=157), males contributed 55% of the deaths and the median age for deaths was 65 years (Q1=49, Q3=79). The median duration of admission for deceased patients was 2 days (Q1=0, Q3=7). We found that 11.3% of admitted patients were vaccinated, and the most common vaccine was Sinopharm (38%). We found that Chinhoyi Provincial Hospital (CPH) (41.8%), Queen Mary (29.2%) and Karoi District Hospital (14.1%) had admitted most patients. The pattern was similar in the absolute number of deceased patients, where CPH contributed 51%, followed by Queen Mary (15%) and Karoi (12%). However, when considering the facility case fatality rate Chegutu had the highest at 90%, followed by Kariba (57%) and Sanyati Baptist (50%). Makonde Christian and St Micheal's Hospitals had a 0% case fatality rate (Table 1)

**Table 1:** demographic characteristics of COVID-19 patients admitted to isolation centres in Mashonaland West, April 2020-2022

Characteristic	Total Admissions n=673	Deceased (n=157) (%)	Discharged (n=516) (%)	p-value
Median age (Years)	40.0 [Q1=25, Q3=65]	65 [Q1=49, Q3=79]	33.0 [Q1=23, Q3=53]	<0.001
Duration of stay (Days)	3.0 [Q1=1, Q3=7]	2.0 [Q1=0, Q3=6]	3.0 [Q1=1, Q3=7]	<0.001
**Sex**				
Female	446 (66.4%)	71 (45%)	375 (73%)	<0.001
Male	227 (33.6%)	86 (55%)	141 (27%)	<0.001
**Vaccination status**				
Not vaccinated	603 (89.7%)	138 (88%)	420 (90%)	0.385
Received at least one dose	70 (11.3%)	19 (12%)	51 (10%)	
**Type of vaccine**				
Sinopharm		14 (20%)	25 (35.8)	0.201
Sinovac		4 (6%)	9 (12.9%)	
Covaxin		0 (0%)	1 (1%)	

**Disease characteristics:** of the 672 admitted patients, 50.5% had mild COVID-19, 21.3% had moderate COVID-19, and 28.2% had severe COVID-19 diagnosed on discharge. Of the patients with mild, moderate and severe COVID-19, 7.9%, 17.2% and 58.5% lost their lives. In addition, patients presented with shortness of breath (38.4%), dry cough (34.9%), headache (30.7%), chest pain (29.6%), general body weakness (24.1%) and fever (19.8%). The most common comorbidity patients presented with was hypertension (28.5%), followed by diabetes (13.8%), HIV (9.7%), chronic pulmonary disease (6.8%), and cardiac disease (5.5%). Only 1.8% of patients had no comorbidities. We found that out of patients diagnosed with hypertension, 44.0% deceased. The moralities for other comorbidities (diabetes, HIV, chronic pulmonary disease) were 42.4%, 57.0% and 69.2%, respectively. Of those with no comorbidities, 38.5% died, and out of the 193 patients who were pregnant, only 3% deceased.

**Duration of stay:** we found that 22.4% of patients were admitted for less than one day. Out of these, 4.4% of patients were admitted for less than one hour and 4.7% of patients were admitted for between 1 hour to 12 hours and 13.3% were admitted for 12-24 hours. Nearly 23% of patients were admitted for 24-48 hours days and 11.9% were admitted for 48-72 hours. The patients who were admitted for more than one week were 6.7% for 8-14 days, 5.5% for 14-28 days and 4.3% for more than 28 days. We found that median time of admission for discharged patients was 1 (Q1=0, Q3=4) days and for those who deceased was 0 (Q1=0, Q3=2) days. We found that the median time the deceased patients spent 4 (Q1=0, Q3=7) days at home before presenting to the health center whilst the median time discharged patients presented was 0 (Q1=0, Q3=3) days. The results of the Kaplan-Meier survival analysis showed that the unadjusted mortality probability was higher in patients admitted for a shorter duration (4 days) than those admitted for longer (9 days) and that, according to the log-rank test, this difference was highly statistically significant (p < 0.001).

**Examination findings (n=672):** we found that 29.6% of patients had low oxygen saturation in the ward, while 16.1% did not have any oxygen saturation done at all. Only 13.1% of patients had pyrexia, whilst 18.5% and 14.3% of admitted patients had tachypnea and tachycardia, respectively. We found that 29.2% were hypertensive in the ward and 11.2% of patients had hypoglycemia (1.8%) and hyperglycemia (9.4%). We found that 43.4% of patients did not have any glucometer done in the ward.

**Clinical management (n=672):** we found that the drugs administered were ceftriaxone (52.4%), paracetamol (51.6%), dexamethasone (36.5%), and zinc sulphate (36.5%). Seventy-two percent of patients were on free air at admission and only 21.8% were given oxygen per face mask and 4.8% were given oxygen via rebreathe bag. No patients in the province were put on ventilators. On admission, 54.2% of patients did not receive any supplementary oxygen and 21.1% of patients were put on oxygen per face masks. Only 4.2% of patients were put on positive pressure oxygen. The complications that developed in the ward were pneumonia (12.4%), acute kidney injury (7.3%), and cerebral vascular accident (7.0%). Most patients (87.6%) did not have any documented complications.

**Pregnant patients:** the cesarean section rate for the admitted 193 pregnant women in the province was 21.8% and 61.7% of them delivered vaginally, and the rest did not deliver in the ward. The outcomes of newborns were generally good with only 1.0% of live birth having poor Apgar's and only 2.6% of deliveries were fresh stillbirths. The province recorded 7 maternal deaths and the maternal mortality ratio during the two years was 2188 per 100 000 live births. Other complications were pregnancy-induced hypertension (PIH) (25.4%), puerperal sepsis (4.1%) and postpartum hemorrhage (PPH).

**Bivariate analysis:** we found that the demographic factors associated with mortality were old age OR 1.08 (95% CI 1.01-1.13), delaying seeking care OR 1.12 (95% CI 1.03-1.21) and being female was protective OR 0.36 (95% CI: 0.18-0.61). In addition, we found the longer the patient was admitted OR 0.99 (95% CI: 0.95-1.11), the less likely the patient was to die. However, this factor was not statistically significant. On signs and symptoms, we found having one of these signs and symptoms individually; fever OR 6.4 (95% CI: 4.3-9.7), shortness of breath OR 5.1 (95% CI 3.4-7.3), dry cough OR 3.0 (95% CI: 2.2-4.8) and headache OR 1.9 (95% CI: 1.8-2.6) was associated with COVID-19 mortality. However, we found that having these four or more symptoms significantly increased mortality risk OR 2.6 (95% CI: 1.8-4.5).

**Independent factors:** we adjusted for factors such as age, sex, discharging diagnosis and multiple comorbidities. The independent comorbidities that were associated with mortality individually were cardiac disease aOR 13.3 (95% CI: 6.1-27.7), diabetes aOR 6.0 (95% CI: 3.8-9.2) and hypertension aOR 4.5 0 (95% CI: 2.8-6.5). When combined, hypertension and diabetes were independent risk factors for mortality aOR 3.4 (95% CI: 1.8-12.1). The independent examination findings associated with mortality were having an abnormal temperature reading aOR 9.5 (95% CI: 6.1-14.9) and having an oxygen saturation below 90% aOR 9.6 (95% CI: 5.9-15.8). The independent clinical management factors associated with mortality were Ivermectin aOR (95% CI: 1.2-37.0), Dexamethasone aOR 2.4 (95% CI: 1.6-3.4) and Heparin/Clexane aOR 1.6 (95% CI: 1.1-2.2). We found that women who were pregnant aOR 0.06 (95% CI: 0.02-0.14) and patients who were put on Azithromycin aOR 0.60 (95% CI: 0.40-0.98), Vitamin C aOR 0.48 (95% CI: 0.31-0.71) and Amoxicillin 0.24 (95% CI: 0.11-0.52) were less likely to die. We also found that putting the patients on oxygen per face mask aOR 0.14 (95% CI: 0.10-0.19) was an independent protective facto (Table 2).

**Table 2:** independent factors associated with COVID-19 mortality, Mashonaland West, 2020-2022

Factor	Crude Odds Ratio	Crude 95% confidence interval	Adjusted odds ratio	95% confidence interval
Age	1.05	1.04-1.06	1.04	1.03-1.05
Female	0.32	0.24-0.47	0.31	0.21-0.46
Four or more symptoms	2.6	1.8-4.5	2.4	1.9-4.4
Heparin/Clexane	1.6	1.1-2.3	1.5	1.1-2.2
Dexamethasone	2.4	1.5-3.9	2.3	1.6-3.4
Amoxicillin	0.24	0.11-0.52	0.23	0.10-0.50
Azithromycin	0.61	0.41 0.90	0.60	0.40-0.98
Vitamin C	0.48	0.36-0.72	0.47	0.31-0.71
Diabetes	6.0	3.2-9.7	5.9	3.8-9.2
Hypertension	4.5	3.1-6.9	4.3	2.8-6.5
Pregnancy	0.07	0.03-0.15	0.06	0.02-0.14
HIV	2.7	1.6-4.8	2.6	1.4-4.6
Oxygen per face mask	0.15	0.09-0.21	0.14	0.10-0.19

**Key informant interviews:** we interviewed key informants in the isolation centers such as the senior health staff in each isolation center, the district health executive and the provincial health executive. Key informants reported that elderly patients had low trust in the public health sector and hence often presented late after exhausting all traditional medicine approaches. Across all isolation centers, staff reported shortages of key medicines such as oxygen and dexamethasone and often vital equipment such as pulse oximeters and glucometers were also in short supply.

## Discussion

We conducted a descriptive analytical audit of the COVID-19 patients admitted in Mashonaland West Province. The study aimed to determine the individual-level characteristics associated with mortality risk in patients admitted to isolation centers around the province. Being old and male were found to be independent risk factors for mortality, which is similar to findings from other African [19] and international [20] studies in which being male among hospitalized patients was associated with a higher risk of mortality while receiving care [21]. This finding could be attributable to men's lower health-seeking behavior [21] and their higher risk of comorbidities as they age. Similar to research by Najera *et al*. in Mexico, patients in our study were only in the hospital for a brief time before passing away [20]. In contrast, research in Italy indicated that an extended stay in the hospital was a predictor of hospital COVID-19 mortality [22]. That these patients were more likely to arrive late at the hospital could explain our findings. This necessitates more research with a bigger sample size to ensure that length of stay has no bearing on death in COVID-19 patients. Patients who had two or more of the symptoms of fever, shortness of breath, headache, or dry cough had a higher risk of dying. Bertsimas *et al*. also found something comparable in international multicentre research [9]. Patients who had a higher level of institutional trust were more likely to meet COVID-19 requirements [23] and therefore were more likely to survive, according to Oksanen *et al*. in Norway, whereas persons who distrusted health institutions were more likely to arrive late, according to Najera *et al*. [20].

We found that patients with comorbidities like HIV, diabetes, and hypertension were also more likely to die. When diabetes was paired with additional comorbidities like hypertension, the chance of death increased. Similarly, the combination of COVID-19 with several comorbidities enhanced the risk of death, according to Najera *et al*. [20]. Several studies have found that people of African heritage are more likely to develop hypertension [11,13] and that hospitalized patients with hypertension in sub-Saharan Africa were twice as likely to die from COVID-19 infection [24]. This could be because the patient's traits that made him more likely to die looked to have a physiologic link to severe COVID-19 disease [2,5,11,19,20,25]. According to a study by Ashish *et al*. in Nepal, there was a reduction in institutional deliveries in the country related to heightened disease transmission fears [26]. However, contrary to both Ashish *et al*. and Westgren *et al*. findings that increased the risk of severe disease among pregnant women [27], we found that pregnant women were more likely to survive. This finding could be attributable to the fact that pregnant and hospitalized patients have more nursing and midwifery personnel available. This, however, requires further research with an even larger sample size to ensure that pregnancy has no role in increasing mortality in patients with COVID-19. Low blood oxygen saturation is one of COVID-19's characteristics, which occurs in most symptomatic individuals [6]. We found that patients with anomalous temperature readings and oxygen saturation were more likely to die. Tracking a patient's saturation and providing oxygen via a face mask to patients with saturations less than 90% reduced the risk of death. This finding is comparable to Abate *et al*. who discovered that delaying the initiation of oxygen therapy increased mortality in patients hospitalized in the Intensive care unit (ICU) [10].

One of the significant causes identified in COVID-19 mortality was the lack of adequate hospital beds and ventilators [9]. In Western countries, the reaction to the COVID-19 pandemic has been to increase hospital capacity and provide more intensive care units (ICUs) and more ventilators. However, although putting patients on oxygen per face mask was protective, all isolation centers in Mashonaland West did not put any patient on a ventilator due to inadequate ventilators and lack of training. This finding is similar to studies in sub-Saharan Africa (SSA) where a shortage of oxygen in health centers was widespread [28,29]. COVID-19 has been linked to coagulopathies in several studies [22,30-32]. However, contrary to the findings of Meizlish *et al*. who found that putting patients on anticoagulants prophylactically reduced mortality in hospitalized patients [31], we found that the use of anticoagulants increased the risk of death in Mashonaland West. We also found that using steroids made patients more likely to die in patients with heart problems, contrary to the findings of research by Rath *et al*. [30]. Our results could be explained by the fact that comorbidities and other risk factors often play a combined role in predicting a patient's death [20,26,33]. However, more research with bigger sample size is needed to confirm that steroids and anticoagulation play a role in increasing mortality in COVID-19 patients.

**Limitations:** this research is not without flaws. We had no control over the sample or the data sources because the data was sourced from publicly available patient notes and audit forms. In addition, because our research was cross-sectional, we could not draw any conclusions about the temporal sequence of events. Despite these obstacles, we used solid statistical analytic approaches to reduce the risk of bias.

## Conclusion

This study shows that COVID-19 mortality risk sharply increased in patients who were older, male and had comorbid diseases such as HIV, diabetes, hypertension, and CVDs in Mashonaland West. However, our study shows that death risk was less for pregnant patients and patients who were put on oxygen and Vitamins C. The study of the source of these variations in risk across patients is central to understanding the impact of differences in individuals' mortality.

### What is known about this topic

- *Approximately 15% of patients develop signs and symptoms severe enough to warrant admission;*
- *The factors that are associated with COVID-19 mortality are comorbidities such as diabetes and hypertension*.

### What this study adds

- *Patients with comorbidities such as hypertension and diabetes were more likely to die; however, pregnant women and those on Vitamin C were independent protective factors; however, there is a need for more research on this topic to certify this*.
